# Understanding factors influencing HPV vaccine uptake among caregivers in Kwara State, Nigeria: A qualitative study

**DOI:** 10.1371/journal.pone.0351659

**Published:** 2026-06-16

**Authors:** Abdulmujeeb Opeyemi Muhammad-Olodo, Laura Asher

**Affiliations:** 1 Nottingham Centre for Public Health and Epidemiology, School of Medicine, University of Nottingham, United Kingdom; 2 Department of Medicine and Surgery, College of Health Sciences, University of Ilorin, Ilorin, Kwara State, Nigeria; Federal University Otuoke, NIGERIA

## Abstract

**Introduction:**

Human papillomavirus (HPV) vaccine prevents over 90% of cervical cancers. In October 2023, Nigeria launched a free HPV vaccination campaign targeting girls aged 9–14 years. Despite removing cost barriers, misinformation about fertility impacts and population control contributed to variable uptake across states. Understanding caregiver decision-making is crucial for improving coverage. This study aimed to explore factors influencing caregivers’ HPV vaccination decisions during Nigeria’s 2023 campaign in Ilorin East Local Government Area, Kwara State.

**Methods:**

A qualitative study using focus group discussions (FGDs) was conducted using purposive and snowball sampling. We recruited 41 caregivers (mean age 47 years; 71% female) of eligible girls from urban and rural communities. Five FGDs were conducted: four with vaccine acceptors (n = 35) and one with decliners (n = 6). Discussions were conducted in Yoruba, audio-recorded, transcribed verbatim, and analysed using Braun and Clarke’s reflexive thematic analysis. Ethical approval was obtained from two institutional review boards.

**Results:**

Four themes emerged from the analysis. Trust operated at multiple levels: institutional (government programmes), interpersonal (healthcare worker competence), and community (religious/traditional leader endorsement). Historical medical mistrust, intensified by COVID-19 experiences, may have manifested as fertility and population control fears. Personal cancer experiences strongly motivated acceptance, whilst concerns about childhood sexuality influenced timing preferences. Despite free provision, barriers included geographic inequities (remote Fulani-Hausa communities were excluded), language barriers (no Hausa translators), school-based delivery gaps, and indirect costs (transport, time). Caregivers recommended house-to-house campaigns, multilingual services, traditional leader engagement, and permanent vaccination centres.

**Conclusion:**

Free vaccine provision is necessary but not sufficient to ensure uptake. Successful HPV vaccination requires rebuilding trust through community engagement, addressing historical medical exploitation concerns, and ensuring equitable access. Integrating these findings into Nigeria’s National Programme on Immunisation could improve coverage from current estimates of 54% to targeted 90%, protecting more girls from cervical cancer whilst respecting community values.

## Introduction

Cervical cancer is the fourth leading cause of death amongst women globally, with more than 342,000 deaths in 2020 [1]. Nigeria has the highest cervical cancer mortality rate in sub-Saharan Africa, with more than 8000 women dying from this disease in 2020 [2]. Since the discovery of Human Papillomavirus (HPV) as a cause of cervical cancer, HPV vaccination has emerged as a crucial preventive measure, with HPV vaccines such as Gardasil-9 preventing more than 90% of cases [3].

Before 2023, the high cost of HPV vaccines in Nigeria posed significant access barriers, particularly for individuals from lower socioeconomic backgrounds [4]. This disparity in access represented a critical healthcare inequity in cervical cancer prevention. To address this inequity, in October 2023 the Federal Government of Nigeria, through the Ministry of Health and National Primary Health Care Development Agency (NPHCDA), partnered with multilateral organisations including the Global Alliance for Vaccines and Immunization (Gavi), International Finance Facility for Immunisation (IFFIm), and World Health Organization (WHO) to launch a nationwide HPV vaccination campaign targeting female children aged 9–14 years [2]. Crucially, the vaccine was made free to access. However, there were reports that the campaign was met with public hesitancy; rumours included claims that the vaccine would cause infertility or premature death [5–7].

Although no official data from the government has been released on the overall uptake of the vaccine, one study shows only 53.9% vaccination uptake amongst adolescent girls in some states [8]. Caregivers are the primary decision-makers in any adolescent vaccination programme. Understanding what influenced caregivers to either accept or decline the HPV vaccine, particularly in the context of misinformation, is crucial to addressing this low response rate in order to improve future vaccination campaigns in Nigeria. This is particularly urgent in states like Kwara, which was selected for Phase 1 implementation of the campaign [9], as lessons learned from early adopter states can inform national rollout strategies. Within Kwara State, Ilorin East Local Government Area (LGA) represents a diverse demography encompassing urban and rural settlements with varied socioeconomic and educational backgrounds. This diversity allows for a comprehensive understanding of factors influencing vaccination decisions across different population segments.

The aim of this study was to explore factors influencing caregivers’ decisions regarding HPV vaccination for their children following Nigeria’s 2023 national vaccination campaign in Ilorin East LGA, Kwara State.

## Methods

### Study design

This study was underpinned by the constructionist paradigm, which posits that individual experiences and decision-making are shaped by shared understanding and interactions within social context [10]. We therefore focused on how shared social meanings, rather than individual psychological traits, shaped vaccination decisions, allowing us to gain understanding of participants’ experiences and the thought-process behind the decisions they took. Focus group discussions (FGDs) were considered most appropriate for gaining rich insights into the community setting because they allow for interaction between participants and facilitate identification of shared community norms and group-level meanings, in addition to revealing diverse perspectives within the dynamics of the group.

Ethical approval for the study was granted by the University of Nottingham Faculty of Medicine and Health Sciences Research Ethics Committee (Reference: FMHS 112–0325) and the University of Ilorin Teaching Hospital (UITH) Research Ethics Committee (Reference: NHREC/02/05/2010).

### Setting

The study was set in Ilorin East LGA, Kwara State, Nigeria. Ilorin is the capital of Kwara State; it is located in the North Central geopolitical zone of Nigeria. Ilorin East is one of three LGAs, and is mainly urban with some rural areas. The main ethnic group in the city is Yoruba, with pockets of Fulani-Hausa settlements in more remote areas. The majority of the Yoruba population work in white and blue-collar jobs while the Fulani-Hausas typically engage in pastoral farming. A mixture of rural and urban areas were used in this study to give a balanced perspective on the campaign. The rural areas were Panada and Oke-Oyi, while the urban areas were Sabo-Oke, Isale Koko and Alalubosa.

### Participants

The study population comprised 41 caregivers of female children aged 9–14 years who were eligible for HPV vaccination during the 2023 national campaign. Caregivers included any adult with primary responsibility for an eligible child (biological parents, grandparents, aunts/uncles, foster parents, guardians). All participants resided in Ilorin East LGA, could communicate in Yoruba, and were involved in the vaccination decision. Those whose children received HPV vaccination before 2023 or who could not recall their decision-making experience were excluded.

Recruitment employed a combination of purposive and snowball sampling techniques. The recruitment took place between 19 May 2025 and 23 May 2025 and was coordinated by a male research assistant in collaboration with community mobilizers familiar with the areas. The community mobilizers were lay community members nominated by local community leaders (Baales and ward heads) for their familiarity with residents and their existing relationships within places of worship and markets; they were not health workers and received a brief verbal orientation about the study’s purpose, eligibility criteria and the importance of recruiting both acceptors and decliners. Mobilizers helped identify caregivers known to have either accepted or declined vaccination during the campaign and made initial introductions, after which the research assistant explained the study and confirmed eligibility. Approximately 60 caregivers were approached across the five communities. Of these, 41 agreed to participate (response rate ≈ 68%); the remainder either could not attend the scheduled session due to work or domestic commitments, or declined to take part without giving a specific reason. Recruitment of acceptors was relatively straightforward, whereas decliners were more difficult to identify, which we revisit in the Strengths and Limitations section. Recruitment was facilitated through community gathering places (places of worship, markets, community centres) and WhatsApp group platforms where recruitment information was shared. Potential participants were given verbal and written information explaining the research in its entirety, before providing written informed consent to participate.

### Data Collection

We held four FGDs with accepting caregivers (two urban, two rural) and one FGD with declining caregivers; the decliner FGD was conducted in an urban setting only, as we were unable to recruit a sufficient number of decliners from rural areas to form a separate group. There were between five to 10 participants per FGD. The FGDs were conducted by a male native Yoruba-speaking research assistant with extensive previous experience of qualitative data collection, to ensure cultural and linguistic appropriateness. FGDs lasted an average of 40 minutes. The FGD guide covered key topics around knowledge and information sources about HPV vaccines, factors influencing acceptance or rejection, and recommendations for improving future vaccination programmes (S1 File). FGDs were held at central community meeting points accessible to participants. All discussions were conducted in Yoruba, the predominant local language, and were intermixed with English, and were audio-recorded with participants’ consent. Participants received ₦2,500 Nigerian Naira (equivalent to approximately £5 at the time of the study) as compensation for their time and transportation costs. This amount was reviewed and approved by the ethics committees as appropriate compensation that did not constitute undue inducement, given local transport costs and the typical 60–90 minute time commitment required of participants.

### Data analysis

FGD audio recordings were transcribed and translated to English. Analysis of the data was done in accordance with Braun and Clarke’s six steps of reflexive thematic analysis [11,12] and NVivo 15 software (*Lumivero, version 15*) [13] was used to manage the data. Familiarisation with the data began with repeated reading of transcripts and simultaneously checking against audio recordings for accuracy. We used inductive coding, with themes developed iteratively [11]. Saturation was assessed during analysis and was considered reached when three consecutive FGDs yielded no new codes; on this basis no additional acceptor FGDs were conducted, although we acknowledge that saturation for decliner perspectives was almost certainly not achieved given that only one decliner FGD was conducted (see Strengths and Limitations). Codes were merged and renamed and codes that shared similarities were eventually grouped into initial overarching themes. Then, themes and sub-themes were reviewed using an iterative approach. In this phase, a more latent approach was taken to uncover deeper meaning behind what was spoken about by participants. Final themes were defined and named such that they not only “tell a story” but also the inter-relationships between themes and sub-themes are made coherent [12]. Illustrative quotes were selected to support themes and sub-themes. Participant identifiers (R01–R10) were assigned within each focus group; the same identifier may therefore refer to different individuals across different FGDs, and full contextual descriptors (sex, urban/rural residence, acceptor/decliner status) are provided alongside every quotation to ensure unambiguous attribution.

## Results

### Study participants

Five FGDs were conducted with 41 caregivers in total; 12 were male and 29 were female. The mean age of participants was 47 years (Table 1). Most participants were married (90.2%) and all were Yoruba ethnicity. Thirty-five (85.0%) participants had accepted vaccination and six (15.0%) participants had declined.

**Table 1:** Socio-demographic characteristics of study participants.

Variable	Frequency	Percentage (%)
**Age**		
< 40 years	11	26.2
40–59 years	22	52.4
≥ 60 years	9	21.4
Mean ± SD	47 ± 12.35	
Total	41	100.0
**Gender**		
Male	12	29.3
Female	29	70.7
Total	41	100.0
**Age of child**		
9 years	4	9.8
10 years	7	17.1
11 years	3	7.3
12 years	3	7.3
13 years	11	26.8
14 years	13	31.7
Total	41	100.0
**Marital status**		
Single	0	0.0
Married	37	90.2
Divorced	2	4.9
Widowed	2	4.9
Total	41	100.0
**Ethnic group**		
Yoruba	41	100.0
**Rural/urban**		
Urban	25	61.0
Rural	16	39.0
Total	41	100.0

*Footnote: Percentages may not sum to 100 due to rounding*

### Overview of themes

Four themes with corresponding sub-themes were identified (Table 2). Each theme is outlined below with supporting quotes, which are labelled: participant identifier, sex, location and acceptor/decliner of vaccine (e.g., R02, F, Rural, Acceptor).

**Table 2:** Themes and sub-themes.

Themes	Subthemes (and section in main text)
**1. Trust as a catalyst or barrier to HPV vaccination**	1.1 Information clarity shapes vaccine awareness 1.2 Varying degrees of confidence in healthcare providers and institutions 1.3 Community-driven trust building 1.4 Distrust fuelled by misinformation and rumours
**2. Community networks and cultural beliefs shape vaccine decisions**	2.1 Past experience of cancer affecting family and friends 2.2 Personal and cultural beliefs shaping perceptions
**3. Perceptions about vaccine safety drive its acceptance**	3.1 Varied concerns about side effects and harm 3.2 Perceived health benefits beyond cancer prevention
**4. Systemic and logistical challenges influence success of vaccination programme**	4.1 Equity gaps in programme implementation 4.2 Government leadership in vaccination programme sustainability 4.3 Lack of free time, transport, and financial constraints impede motivation to vaccinate

### Theme 1: Trust as a catalyst or barrier to HPV vaccination

Trust emerged as a pivotal factor influencing vaccination decisions, operating at multiple levels—from how information was received to faith in vaccinators’ competence.

#### Subtheme 1.1: Information clarity shapes vaccine awareness.

Participants’ awareness of the vaccination programme originated from multiple channels—children relaying information received at school, radio, social media, and personal discussions. While radio and social media provided broad reach, some found it insufficient: *“I heard on [the] radio and my daughter also heard in school, but the information was not enough, because we don’t know what might likely occur if she takes the vaccine”* (R02, F, Urban, Decliner). Personal communications proved more influential: *“I heard it over the phone. One of my friends called me to say they are bringing this particular vaccine, this is what it does, let your child take it”* (R03, M, Rural, Acceptor).

#### Subtheme 1.2: Varying degrees of confidence in healthcare providers and institutions.

Trust in government programmes and vaccinator competence significantly influenced acceptance. One participant who had assisted the vaccination team stated: *“We know that the government will not bring what will harm the public, and will not bring in diseases… In fact, I’ve been working with them for more than a month, and I know it is a health programme”* (R10, M, Rural, Acceptor). The apparent competence of vaccination personnel significantly influenced acceptance. Those who perceived vaccinators as qualified were more willing to accept: *“What made it easy for us to decide on vaccination was knowing that those that were employed on this job, we know they are capable. It was trained officers that were employed”* (R06, F, Rural, Acceptor). However, encounters with inadequately trained personnel fostered mistrust: *“They need to enlighten their workers, because sometimes when you ask them question, they will not be able to respond… maybe they didn’t give them proper training or education”* (R03, F, Urban, Decliner). This account illustrates how perceived competence of vaccinators served as a proximal cue for the broader trustworthiness of the health system: when caregivers felt their questions were not adequately answered, doubts about vaccinator training appeared to generalise to doubts about the vaccine itself and the institutions delivering it.

#### Subtheme 1.3: Community-driven trust building.

Participants emphasised the crucial role of community leaders and local networks in building trust. One participant advocated for indigenous involvement: *“When they [government] want to carry out the programme, they should make sure that an indigene of the community is involved, to mobilise the people… Whenever they [community members] see one of their own among visitors, they cooperate better”* (R10, M, Rural, Acceptor). Religious leaders’ endorsement carried weight: *“I am the pastor of the church of Panada, and we have brought this programme, because we know that this will benefit us”* (R03, M, Rural, Acceptor). Similarly, healthcare professionals within social networks provided trusted guidance: *“My sister called me who is a nurse and explained to me…. that was why I allowed the child to take the vaccine”* (R04, F, Urban, Acceptor).

#### Subtheme 1.4: Distrust fuelled by misinformation and rumours.

Widespread misinformation claiming the vaccine would be used to control population and reduce fertility significantly undermined trust amongst some participants: *“…some people [online] came with a rumour that maybe the whites are trying to use it to reduce our population”* (R01, M, Rural, Acceptor). One decliner explicitly linked her hesitancy about the HPV vaccine to her experience during the COVID-19 vaccination campaign, reporting that *“during COVID-19, they told us to collect the vaccine but later we heard that it was because of our population that was why the vaccine was introduced… even some vaccine that they brought, I did not allow them to take it”* (R01, F, Urban, Decliner). This illustrates how earlier vaccine experiences appeared to shape contemporary decisions through accumulated rumours about the underlying purpose of mass immunisation programmes. Systemic healthcare failures further eroded trust, with one participant suspecting the vaccines being given were of poor quality: *“I thought they were giving vaccines that were almost expired in public hospitals, while the good ones are being administered at private hospital”* (R03, F, Urban, Decliner).

### Theme 2: Community networks and cultural beliefs shape vaccine decisions

#### Subtheme 2.1: Past experience of cancer affecting family and friends.

Personal encounters with cervical cancer sometimes powerfully motivated acceptance. One widower recounted: *“My late wife died of cancer, so immediately I got the information, I said I wouldn’t want to mess up again”* (R04, M, Urban, Acceptor).

#### Subtheme 2.2: Personal and cultural beliefs shaping perceptions.

Beliefs about childhood sexual exposure as a prerequisite for vaccination significantly influenced vaccination timing preferences. One mother articulated: *“They are still children, I prefer to educate my child on pros and cons of sexual purity… before she is exposed [to the vaccine]”* (R03, F, Urban, Decliner). This sentiment reflected deeper concerns about age-appropriateness as another declared: *“I did not allow her to take the vaccine when they said it is cervical cancer; these children are young, they are not sexually active”* (R03, F, Urban, Decliner). Conversely, some participants viewed vaccination as protection for future fertility: *“…if I did not allow her to take the vaccine, when she grows up, she might have difficulties in childbearing”* (R04, F, Urban, Acceptor).

### Theme 3: Perceptions about vaccine safety drive its acceptance

#### Subtheme 3.1: Varied concerns about side effects and harm.

Participants’ interpretations of side effects varied dramatically, influencing their vaccination decisions. Some normalised mild reactions that resolved a few days following the vaccination, whilst others dismissed concerns entirely: *“There has been no problem with our children, since they got vaccinated and they took paracetamol”* (R08, F, Rural, Acceptor). However, inadequate pre-vaccination counselling about potential side effects deterred others, who explicitly attributed their refusal to a perceived lack of explanation about possible adverse effects: *“She did not take the vaccine, because they did not explain the implications, the side effect of the vaccine”* (R04, M, Urban, Decliner).

#### Subtheme 3.2: Perceived health benefits beyond cancer prevention.

Some participants attributed additional health improvements to the vaccine, beyond scientific reasoning: *“People even commented that when they got vaccinated, they notice a difference in their [children’s] bodies, because they used to have a high temperature before but now, they don’t”* (R09, F, Rural, Acceptor).

### Theme 4: Systemic and logistical challenges shape success of the vaccination programme

#### Subtheme 4.1: Equity gaps in programme implementation.

Geographic and linguistic barriers seemed to prevent equitable vaccine distribution. Participants highlighted the exclusion of remote Fulani-Hausa communities: *“I want the government to encourage the less educated in the hinterlands… It does not cost them anything o, but they won’t take it”* (R02, F, Rural, Acceptor). Language barriers further marginalised these groups: *“If these people want to go to the Fulanis, they should have someone that understands Hausa… but if you don’t understand their language, they will run and take their child away”* (R03, M, Rural, Acceptor). Additionally, it was recommended that the government provide sign language interpreters for those who are deaf or other tailored support for those who have special needs, so all members of the community could access vital information and benefit equally from public health services. School-based delivery created additional gaps, with some schools reportedly overlooked entirely while others received inadequate notice. A teacher amongst the participants noted: *“Last year… there were some schools that didn’t receive letters, they [vaccinators] will just come suddenly with the vaccine… the parents said since they were not informed… they won’t allow their children to take it”* (R05, F, Urban, Acceptor). Participants recommended that schools be given written advance notice (at least one to two weeks) and that these notices should be sent home with students for parents to be made aware before vaccination day, to address these communication gaps.

#### Subtheme 4.2: Government leadership in vaccination programme sustainability.

Participants emphasised the need for sustained government commitment beyond initial campaigns: *“What I want to add is that… follow-up is the problem. Most times the government stop in the middle, and then we will not see it again or hear about it again”* (R06, M, Rural, Acceptor). Participants recommended integrating HPV vaccination into existing healthcare infrastructure for sustainability. One suggested: *“I will prefer going to the hospital to take the vaccine than allowing someone random to administer the vaccine to children”* (R03, F, Urban, Decliner). Private sector engagement was also advocated: *“Government should look for qualified private hospitals to administer the vaccines”* (R06, F, Rural, Acceptor).

#### Subtheme 4.3: Lack of free time, transport, and financial constraints impede motivation to vaccinate.

Despite free vaccine provision, indirect costs posed barriers. Transportation expenses particularly burdened those far from fixed-post sites. House-to-house delivery emerged as the preferred solution to address both access and awareness gaps: *“I will say it would have been better if they moved from house to house, because the school they are going to, it’s not all the children that will get it there”* (R02, F, Rural, Acceptor).

## Discussion

This study revealed that while multiple information sources created awareness of the HPV vaccination campaign, personal communication proved more influential than mass media. Trust emerged as the central determinant of vaccination decisions, operating through confidence in government programmes, vaccinator competence, and community endorsement, while historical medical mistrust and COVID-19 experiences appeared to create substantial barriers through fears of population control and fertility impacts. Personal experiences with cervical cancer deaths served as powerful motivators for acceptance, though personal beliefs about childhood sexual exposure influenced timing preferences. Despite free vaccine provision, systemic barriers including geographic inequities, school-based delivery gaps, transportation costs, and programme discontinuity limited access, particularly for remote and marginalised communities. Caregivers recommended house-to-house delivery, healthcare infrastructure integration, multilingual communication strategies, and sustained government commitment to improve future vaccination programmes.

This study identified multiple information channels that shaped caregivers’ awareness and decisions about HPV vaccination. Radio emerged as the primary mass media source, consistent with previous research highlighting mass media’s effectiveness for health messaging in African contexts [14]. This is particularly relevant for intervention design in Nigeria, where radio coverage in rural areas typically exceeds household internet and social media access, making radio a more equitable channel for reaching geographically dispersed and lower-literacy populations than digital platforms. Social media was associated with a propensity for misinformation. This finding aligns with evidence that social media can simultaneously increase awareness while spreading misinformation that undermines vaccination programmes [15]. Notably, school children served as important information conduits to their caregivers, aligning with previous research findings proposing the school-based programmes as an important model for mass vaccination campaigns [16].

Trust emerged as the pivotal factor determining vaccine acceptance or rejection. This operated at multiple levels: institutional trust in government programmes, interpersonal trust in healthcare workers’ competence, and community trust through religious and social networks. Caregivers who expressed confidence in government intentions and perceived vaccinators as qualified were more likely to accept vaccination. This finding corroborates previous research showing that trust in healthcare systems significantly influences vaccine uptake in sub-Saharan Africa [17]. The salience of trust in our findings aligns closely with the ‘3C’ model of vaccine hesitancy (confidence, complacency and convenience), where the trust dimensions described here map most directly onto the ‘confidence’ component—that is, trust in the vaccine, in the system delivering it, and in the policymakers who decide on it—while geographic and indirect cost barriers (Subthemes 4.1 and 4.3) reflect the ‘convenience’ component.

Conversely, historical medical mistrust appeared to manifest through fears that the vaccine was intended to control population levels through reducing fertility, echoing documented concerns about Western medical interventions in African contexts [18]. A broader context of unethical medical experimentation has been documented in the literature—ranging from German-backed sterilisation tests on Herero women in the 1900s to the Pfizer meningitis drug trial in Kano, northern Nigeria, in which eleven children died during testing of an experimental antibiotic in the 1990s [19, 20]. Although none of our participants explicitly named these specific historical events, the recurrence of population-control narratives in our data may help explain why such narratives resonate, as they echo documented historical experiences of medical exploitation in the region. The COVID-19 pandemic appeared to reactivate such concerns, as one decliner explicitly linked her current refusal to rumours encountered during the COVID-19 vaccination roll-out, suggesting that perceived adverse experiences with one vaccination programme may contribute to hesitancy towards subsequent ones [21].

Personal familial experiences with cervical cancer deaths strongly motivated acceptance, aligning with a previous study in Ethiopia where parents that recognised previous family history of cervical cancer as a risk factor were more willing to vaccinate their children [22]. Personal beliefs about appropriate age for vaccination influenced eligibility timing preferences, with some caregivers preferring to delay vaccination until their children attained sexual maturity. These findings corroborate existing literature on the strong dominance of parental authority over adolescent health decision-making in the African context [23,24], a contrast that has been noted with Western settings such as the United Kingdom, where children under 16 may exercise a degree of autonomy over health decisions through the legal principle of Gillick Competence [25].

Caregivers’ experiences revealed significant implementation challenges that limited vaccine access despite free provision. Geographic disparities were prominent, with participants noting that remote communities such as the Fulani-Hausa communities experienced lower coverage due to language barriers and limited outreach. Research shows that these predominantly nomadic communities have Nigeria’s lowest literacy rates, limited access to immunisation and maternal health services, and exclusion from health insurance schemes [26,27]. This marginalisation intersects with linguistic barriers [28], as health information is rarely provided in Fulfulde or Hausa in predominantly Yoruba areas, creating disadvantages that participants recognised when advocating for Hausa-speaking vaccinators.

Furthermore, the school-based delivery model, while efficient for some, excluded children whose schools were overlooked or inadequately notified. This finding supports evidence that school-based programmes require comprehensive planning to ensure equitable coverage [29]. In addition, the lack of programme continuity emerged as a major concern, with caregivers noting that previous health initiatives often started strongly but lacked follow-through. Interestingly, this concern about programme sustainability persists despite the Nigerian government’s recent integration of HPV vaccination into the National Programme on Immunisation schedule, ensuring ongoing availability beyond the initial campaign [30]. This disconnect highlights a critical communication gap, suggesting that policy changes alone are insufficient without corresponding community communication strategies to build awareness and confidence in sustained government commitment.

Caregivers offered recommendations for improvement, including house-to-house delivery to reach underserved communities, vaccine integration into existing healthcare infrastructure for sustainability, and inclusion of private hospitals to expand access points. They emphasised the need for culturally appropriate communication strategies, including translators for minority languages and sign language interpreters for inclusive reach.

### Strengths and limitations

This study presents some strengths. First, the reasonably balanced gender representation of participants (29.3% male, 70.7% female) reflects changing dynamics in reproductive health decision-making. While such decisions have traditionally been dominated by mothers [31], the fair participation of fathers gives a broader understanding of household vaccination dynamics. Second, the study captures real-world decision-making experiences rather than hypothetical intentions. Unlike previous Nigerian studies conducted before vaccine implementation [32,33], this research examined actual choices made during an active campaign, providing more reliable insights into factors that genuinely influence vaccination behaviour when faced with real consequences.

However, several limitations are acknowledged. First, while participants discussed the hesitancy of Fulani-Hausa communities, these insights came second-hand from Yoruba participants rather than direct engagement with these minority groups [28]. Direct inclusion of their voices would have strengthened understanding of their vaccination decisions, particularly given participants’ observations about the need for Hausa-speaking vaccinators.

Secondly, the difficulty in recruiting vaccine-declining caregivers resulted in only one decliner FGD (n = 6), which limited exploration of rejection factors. While the original protocol planned for equal numbers of acceptor and decliner FGDs, recruitment of vaccine-declining caregivers proved challenging, likely reflecting the intensive vaccination efforts by the Kwara State government [9]. Given the small number of decliners (n = 6), our findings on barriers and reasons for refusal should be considered preliminary and require further investigation in larger samples of vaccine-declining caregivers. Thematic saturation for decliner perspectives was almost certainly not achieved, and the single decliner FGD was conducted in an urban setting, so rural decliner perspectives are not represented in our data. The analysis and conclusions in this study therefore primarily reflect acceptor perspectives, with decliner insights presented as exploratory. Future research should specifically oversample decliners using targeted recruitment strategies (e.g., approaching community leaders in areas with documented lower coverage and conducting individual interviews where group settings may inhibit candour about refusal).

Third, social desirability bias is a recognised limitation of this study. In the FGD setting, particularly in the acceptor groups, participants may have been reluctant to express vaccine-hesitant or vaccine-refusing views in front of peers who had vaccinated their children, especially given the broader pro-vaccination climate associated with the Kwara State campaign. This dynamic may also help explain the difficulty in recruiting decliners and the relatively small number of declining caregivers who agreed to take part.

### Implications

Our findings highlight the importance of the Nigerian Ministry of Health and NPHCDA training healthcare workers to provide clear and consistent information about vaccine benefits and expected side-effects. We propose that the National Orientation Agency of Nigeria could develop strategies to counter misinformation, through addressing concerns about a fertility and population control agenda in the context of historical Western medical mistrust. Importantly, any such counter-misinformation messaging should be pre-tested with target communities (for example, through community advisory panels and small-scale message-testing focus groups) prior to large-scale rollout, in order to avoid unintentionally reinforcing the very rumours the messaging seeks to dispel. This could include engaging religious and community leaders as trusted voices in vaccination campaigns, work that could be implemented by the NPHCDA. Furthermore, high-quality research involving co-production with marginalised Fulani-Hausa communities is needed to better understand barriers to access amongst this group and thereby improve equitable access to vaccination; such research should be conducted using qualitative methods in Hausa or Fulfulde by native speakers, ideally co-investigators or community researchers from the same communities, to ensure cultural and linguistic authenticity. Finally, improvements to vaccination programme delivery, such as ensuring all schools and caregivers have sufficient advance notice, could potentially have tangible impacts on vaccine uptake. The Government could also consider adopting the house-to-house approach to vaccination, which was successfully implemented during oral polio vaccination campaigns in the early 2000s, for such a high-priority immunisation initiative [34]. We acknowledge, however, that house-to-house delivery is more resource-intensive than fixed-post or school-based delivery, with substantially higher requirements in terms of trained personnel, transport, cold-chain logistics and supervision. Any decision to scale up this approach should therefore be informed by an explicit assessment of cost, feasibility, and the incremental coverage gain achieved over less intensive delivery models.

## Conclusion

This study provides insights into HPV vaccination experiences in Nigeria, with a focus on understanding actual decision-making processes. The findings reveal that successful vaccination programmes require more than free vaccine provision. By addressing the complex factors influencing caregiver decisions, public health programmes can be designed to be more equitable and sustainable, thus protecting children from preventable diseases while respecting community values and concerns. There is now a need for quantitative studies, both in Kwara State and other Nigerian states, to assess the prevalence of the specific barriers identified here—such as the proportion of caregivers endorsing fertility concerns, perceiving vaccinators as inadequately trained, or facing transport-related barriers—so that future interventions can be appropriately targeted.

## Supporting information

S1 File Focus Group Discusssion guide. Semi-structured guide used to facilitate the five focus group discussions, including probes on knowledge, decision-making, access and recommendations.
(DOCX)

S2 File COREQ checklist. (DOCX)
