# Supplementary material for: Understanding factors influencing HPV vaccine uptake among caregivers in Kwara State, Nigeria: A qualitative study
Source: PLoS One. 2026 Jun 16;21(6):e0351659. doi: 10.1371/journal.pone.0351659 (PMC13271438; doi:10.1371/journal.pone.0351659)
Supplement: S1 File — Semi-structured guide used to facilitate the five focus group discussions, including probes on knowledge, decision-making, access and recommendations. (DOCX) [file pone.0351659.s001.docx]

**Appendix A: FGD discussion guide**

A. Knowledge and Information Sources

1. What do you know about the HPV vaccine?

2. What were your sources of information about HPV vaccines?

Probe: How did you first hear about the vaccination programme?

Probe: Was the information clear and sufficient?

Probe: What additional information would have been helpful?

B. Decision-Making Experience

3. Could you take me through your experience with the HPV vaccination programme?

Probe: Let's start from the beginning

Probe: What thoughts went through your mind?

Probe: Who did you consult?

4. What factors influenced your decision to [accept/decline] the HPV vaccine for your child?

Probe: Can you give specific examples?

Probe: What role did family members play?

Probe: What were your main concerns, if any?

Probe: What made you confident/hesitant about the vaccine?

C. Access and Implementation

5. How did you find the process of getting [or deciding not to get] the vaccine?

Probe: Please tell me about your experience

Probe: What made it easier/difficult?

Probe: Tell me about the vaccination day (for acceptors)

Probe: How was the service delivery?

6. How would you describe the role of cost in your decision?

Probe: How did the free vaccination affect your decision?

Probe: Were there other costs involved (transportation, time off work, etc.)?

D. Support and Barriers

7. For acceptors: What made it easier for you to get your child vaccinated?

For decliners: What would have made you more likely to accept the vaccine?

Probe: What support would have been helpful?

Probe: What challenges did you face?

E. Recommendations and Additional Thoughts

8. What suggestions do you have for improving the vaccination programme?

9. Is there anything else you would like to share about your experience with the HPV vaccination programme?

10. Why would you want to stick to your decision on getting/not getting the vaccine?

Probe: Any other thoughts about how the programme could better serve families?
