# Supplementary material for: Understanding factors influencing HPV vaccine uptake among caregivers in Kwara State, Nigeria: A qualitative study
Source: PLoS One. 2026 Jun 16;21(6):e0351659. doi: 10.1371/journal.pone.0351659 (PMC13271438; doi:10.1371/journal.pone.0351659)
Supplement: Attachment — Submitted filename: Response_to_Reviewers_PLoS_One_LA.docx [file pone.0351659.s003.docx]

**PONE-D-26-17339 – “Understanding factors influencing HPV vaccine uptake among caregivers in Kwara State, Nigeria: A qualitative study”**

**Response to reviewer’s comments**

We are grateful to the reviewer for their thorough and constructive feedback on our manuscript. The comments have helped us substantially strengthen the methodological clarity, balance the framing of our findings, and refine the language and presentation of the paper. We have addressed every point raised below, indicating the relevant line numbers in the revised manuscript where changes have been made. Our amendments and responses are detailed below.

**Major Concerns**

1. Data Availability Statement

**Response**: We thank the reviewer and the editor for highlighting this. We agree that our previous Data Availability Statement was insufficiently specific and we have revised it to comply with PLOS ONE policy and to align the manuscript text with the submission system. Because the qualitative material carries a residual risk of re-identification (small community, distinctive personal narratives), unrestricted public deposition is not appropriate. Anonymised transcripts are however available to qualified researchers upon reasonable request, with access governed by the institutional ethics committee that approved this study. The revised Data Availability Statement now reads:

"The qualitative data generated during this study contain potentially identifying information and cannot be made publicly available, given the residual risk of re-identification arising from the small community in which the study was conducted and the distinctive personal narratives shared by participants. Anonymised transcripts and the FGD guide may be made available to qualified researchers who submit a methodologically sound research proposal and sign a data access agreement prohibiting re-identification and onward sharing. Requests should be directed to the University of Nottingham Faculty of Medicine and Health Sciences Research Ethics Committee (Reference: FMHS 112-0325), which can be contacted at [fmhs-researchethics@nottingham.ac.uk]. The committee, not the authors, will determine whether each request meets the conditions of the original ethical approval."

We have also updated the Data Availability Statement in the submission system so that it matches this manuscript text exactly, and we have changed the system selection to the PLOS option that most closely corresponds to "data are restricted by ethics and available on request through a non-author contact."

2. *Limited Representation of Vaccine Decliners – Potential Bias. The study includes 35 acceptors (four FGDs) and only 6 decliners (one FGD). The manuscript acknowledges this as a limitation but the analysis and discussion treat the findings as broadly representative of factors influencing both acceptance and declination. With such a small and potentially unrepresentative sample of decliners, factors specific to decliners may be under-identified, the single (urban) decliner FGD may not capture rural decliner perspectives, and thematic saturation for decliner perspectives was almost certainly not achieved. The reviewer requested that the manuscript be reframed to emphasise that the findings primarily reflect acceptor perspectives, that a sentence be added in the Discussion stating the decliner findings should be considered preliminary, and that we consider how the title reflects this.*

**Response:** We thank the reviewer for this important point and agree that the framing required adjustment. We have made the following changes:

• In the Strengths and Limitations section, we have added the sentence: “Given the small number of decliners (n=6), our findings on barriers and reasons for refusal should be considered preliminary and require further investigation in larger samples of vaccine-declining caregivers. Thematic saturation for decliner perspectives was almost certainly not achieved, and the single decliner FGD was conducted in an urban setting, so rural decliner perspectives are not represented in our data. The analysis and conclusions in this study therefore primarily reflect acceptor perspectives, with decliner insights presented as exploratory.” (lines 400–404 of the revised manuscript).

• We have also added a sentence on targeted future recruitment strategies for decliners (lines 405–408).

• In the Methods (Data Collection) section we have explicitly stated that “the decliner FGD was conducted in an urban setting only, as we were unable to recruit a sufficient number of decliners from rural areas to form a separate group” (lines 131–132).

After careful consideration, we have decided not to alter the title of the paper. The current title (“Understanding factors influencing HPV vaccine uptake…”) refers to uptake decisions in general, which encompass both acceptance and refusal, and we feel this remains accurate. We believe the explicit reframing in the Limitations and Discussion sections, combined with the new clarifying language at the start of the relevant Methods passages, adequately signals the acceptor focus of the analysis without changing the title.

**3.** *Missing Information on Recruitment and Sampling. Several details are missing: how community mobilizers were selected (health workers, community leaders, lay volunteers); the response rate (how many caregivers were approached, how many declined); and how saturation was determined. Please add a paragraph in Methods describing the recruitment process in more detail and specify how saturation was assessed.*

**Response:** We have substantially expanded the recruitment description in the Methods section. The revised paragraph (lines 112–124) now reads: “The community mobilizers were lay community members nominated by local community leaders (Baales and ward heads) for their familiarity with residents and their existing relationships within places of worship and markets; they were not health workers and received a brief verbal orientation about the study’s purpose, eligibility criteria and the importance of recruiting both acceptors and decliners. Mobilizers helped identify caregivers known to have either accepted or declined vaccination during the campaign and made initial introductions, after which the research assistant explained the study and confirmed eligibility. Approximately 60 caregivers were approached across the five communities. Of these, 41 agreed to participate (response rate ≈ 68%); the remainder either could not attend the scheduled session due to work or domestic commitments, or declined to take part without giving a specific reason. Recruitment of acceptors was relatively straightforward, whereas decliners were more difficult to identify and persuade to attend, which we revisit in the Strengths and Limitations section.”

In the Data analysis section we have also clarified how saturation was assessed: “Saturation was assessed during analysis and was considered reached when three consecutive FGDs yielded no new codes; on this basis no additional acceptor FGDs were conducted, although we acknowledge that saturation for decliner perspectives was almost certainly not achieved given that only one decliner FGD was conducted (see Strengths and Limitations).” (lines 152–156).

**4.** *Overstatement of Findings on “Historical Medical Mistrust”. The manuscript presents historical medical exploitation events (German-backed sterilisation tests, the Pfizer meningitis trial) as directly causative of current HPV vaccine hesitancy without empirical evidence from the participants themselves. No participant explicitly mentioned the Pfizer trial or colonial-era experiments. Please temper the language to reflect that these historical events may contribute to a broader context of mistrust, rather than stating they directly cause current hesitancy. Specifically change “explain why population control narratives resonate so powerfully as they echo real experiences” to “may help explain why population control narratives resonate, as they echo documented historical experiences of medical exploitation.” Ensure that the manuscript does not attribute to participants beliefs or knowledge they did not explicitly express.*

**Response:** We thank the reviewer for this important methodological point and agree that the original wording overstated the causal link. We have rewritten the relevant Discussion paragraph (lines 335–347) to make clear that this is an inferential interpretation rather than a participant-reported attribution. The revised text now reads: “Conversely, historical medical mistrust appeared to manifest through fears that the vaccine was intended to control population levels through reducing fertility, echoing documented concerns about Western medical interventions in African contexts. A broader context of unethical medical experimentation has been documented in the literature—ranging from German-backed sterilisation tests on Herero women in the 1900s to the Pfizer meningitis drug trial in Kano, northern Nigeria, in which eleven children died during testing of an experimental antibiotic in the 1990s. Although none of our participants explicitly named these specific historical events, the recurrence of population-control narratives in our data may help explain why such narratives resonate, as they echo documented historical experiences of medical exploitation in the region. The COVID-19 pandemic appeared to reactivate such concerns, as one decliner explicitly linked her current refusal to rumours encountered during the COVID-19 vaccination roll-out, suggesting that perceived adverse experiences with one vaccination programme may contribute to hesitancy towards subsequent ones.”

We have also softened the related abstract sentence (“may have manifested as fertility and population control fears”, line 31) and the introductory Discussion paragraph (“appeared to create substantial barriers”, line 303), so that all causal claims about historical mistrust are now appropriately hedged.

**Minor Revisions – Abstract**

1. *“resulted in variable uptake across states” – change “resulted in” to “contributed to” (causality cannot be established from this study alone).*

**Response:** We have made this change. The Abstract now reads “…misinformation about fertility impacts and population control contributed to variable uptake across states” (lines 17–18).

2. *“Four themes emerged from analysis” – add “(Table 2)” for clarity.*

**Response:** We have added the table reference: “Four themes emerged from analysis (Table 2)” (line 28).

3. *“free vaccine provision alone is insufficient” – add “but not sufficient” after “free provision.”*

**Response:** We have rephrased the conclusion sentence to: “Free vaccine provision is necessary but not sufficient.” (line 38).

**Minor Revisions – Introduction**

1. *“Cervical cancer is the fourth leading cause of death amongst women globally…” – reference confirmed (Sung et al., 2021).*

**Response:** Acknowledged. The reference is retained (line 46).

2. *“the Federal Government of Nigeria… partnered with Gavi, IFFIm, and WHO” – add the year (2023) earlier in the sentence for clarity.*

**Response:** We have moved the year to the beginning of the sentence: “To address this inequity, in October 2023 the Federal Government of Nigeria, through the Ministry of Health and National Primary Health Care Development Agency (NPHCDA), partnered with multilateral organisations…” (lines 54–56).

3. *“Understanding what influenced caregivers… is crucial to understand this low response rate” – change second “understand” to “addressing.”*

**Response:** This has been amended: “…is crucial to addressing this low response rate in order to improve future vaccination campaigns in Nigeria” (line 66).

**Minor Revisions – Methods**

1. *“This study was underpinned by the constructionist paradigm” – add a sentence on how this paradigm influenced data collection and analysis.*

**Response:** We have added the following sentence to clarify how the paradigm shaped our approach: “We therefore focused on how shared social meanings, rather than individual psychological traits, shaped vaccination decisions, allowing us to gain understanding of participants’ experiences and the thought-process behind the decisions they took.” (lines 83–85).

2. *“Focus group discussions (FGDs) were considered most appropriate” – justify why FGDs rather than individual interviews. Mention that FGDs allow for interaction and identification of shared community norms.*

**Response:** We have expanded this justification: “Focus group discussions (FGDs) were considered most appropriate for gaining rich insights into the community setting because they allow for interaction between participants and facilitate identification of shared community norms and group-level meanings, in addition to revealing diverse perspectives within the dynamics of the group.” (lines 85–89).

3. *Ethical approval – provide approval numbers for both committees (UoN and UITH). Currently missing.*

**Response:** We have added both approval reference numbers: “…granted by the University of Nottingham Faculty of Medicine and Health Sciences Research Ethics Committee (Reference: FMHS 112-0325) and the University of Ilorin Teaching Hospital (UITH) Research Ethics Committee (Reference: NHREC/02/05/2010).” (lines 90–93). The reference numbers are taken from the original ethics approval letters.

4. *Recruitment – add more detail on response rate and mobilizer roles.*

**Response:** This has been substantially expanded – see our response to Major Concern 3 above. The revised text now describes the lay status and orientation of mobilizers, gives the approximate number of caregivers approached (~60), and reports the response rate (≈68%) (lines 111–121).

5. *“We held four FGDs with accepting caregivers… and one FGD with declining caregivers” – specify that the decliner FGD was urban only. Add: “We were unable to recruit decliners from rural areas.”*

**Response:** We have made this explicit: “…the decliner FGD was conducted in an urban setting only, as we were unable to recruit a sufficient number of decliners from rural areas to form a separate group.” (lines 131–132).

6. *“Participants received 2,500 (equivalent to £5) as compensation” – specify the currency (2,500 Nigerian Naira). Add that this amount was ethically approved and did not constitute undue inducement.*

**Response:** We have specified the currency and added the ethical justification: “Participants received ₦2,500 Nigerian Naira (equivalent to approximately £5 at the time of the study) as compensation for their time and transportation costs. This amount was reviewed and approved by the ethics committees as appropriate compensation that did not constitute undue inducement, given local transport costs and the typical 60–90 minute time commitment required of participants.” (lines 141–145).

**Minor Revisions – Results**

1. *Table 1 – add a footnote: “Percentages may not sum to 100 due to rounding.” Verify the “Age of child” frequencies, which sum to 42 not 41.*

**Response:** The corrected "Age of child" frequencies now sum to 41, which is consistent with the total caregiver sample size, and the percentages have been recalculated accordingly. We have also added Total rows for each categorical variable as requested, and added the footnote "Percentages may not sum to 100 due to rounding."

2. *Marital status – add a Total row.*

**Response:** We have added Total rows to all categorical variables in Table 1, including Age, Gender, Age of child, Marital status, and Rural/urban. The percentages have also been recalculated where necessary; the Gender row now correctly reads 12 male (29.3%) and 29 female (70.7%) of the 41 participants.

3. *Theme 1, Subtheme 1.2 – the quote about inadequately trained personnel (R03, F, Urban, Decliner) is important. Add a brief interpretive comment linking this to trust in health system competence.*

**Response:** We have added the following interpretive sentence after the quote: “This account illustrates how perceived competence of vaccinators served as a proximal cue for the broader trustworthiness of the health system: when caregivers felt their questions were not adequately answered, doubts about vaccinator training appeared to generalise to doubts about the vaccine itself and the institutions delivering it.” (lines 202–205).

4. *Subtheme 1.4 – the quote about COVID-19 experiences does not explicitly mention COVID-19. Clarify and ensure the quote is fully presented.*

**Response:** We have replaced the previous summary sentence with a fully presented decliner quote that explicitly mentions COVID-19: “One decliner explicitly linked her hesitancy about the HPV vaccine to her experience during the COVID-19 vaccination campaign, reporting that ‘during COVID-19, they told us to collect the vaccine but later we heard that it was because of our population that was why the vaccine was introduced… even some vaccine that they brought, I did not allow them to take it’ (R01, F, Urban, Decliner). This illustrates how earlier vaccine experiences appeared to shape contemporary decisions through accumulated rumours about the underlying purpose of mass immunisation programmes.” (lines 221–227).

5. *Subtheme 3.1 – “inadequate pre-vaccination counselling about potential side effects deterred others” – add a quote that explicitly mentions lack of counselling. The existing R04 quote is acceptable.*

**Response:** We have retained the existing R04 quote and added a brief connecting clause to make the link to lack of counselling more explicit: “…inadequate pre-vaccination counselling about potential side effects deterred others, who explicitly attributed their refusal to a perceived lack of explanation about possible adverse effects: ‘She did not take the vaccine, because they did not explain the implications, the side effect of the vaccine’ (R04, M, Urban, Decliner).” (lines 251–255).

6. *Subtheme 4.1 – School-based delivery gaps. Add a recommendation from participants about how to improve this (e.g., advance notice, letters sent home with students).*

**Response:** We have added the following participant recommendation at the end of Subtheme 4.1: “Participants recommended that schools be given written advance notice (at least one to two weeks) and that these notices should be sent home with students for parents to be made aware before vaccination day, to address these communication gaps.” (lines 277–279).

**Minor Revisions – Discussion**

1. *“Radio emerged as the primary mass media source” – add that radio reach in rural areas is often higher than internet/social media, which is relevant for intervention design.*

**Response:** We have added the following sentence: “This is particularly relevant for intervention design in Nigeria, where radio coverage in rural areas typically exceeds household internet and social media access, making radio a more equitable channel for reaching geographically dispersed and lower-literacy populations than digital platforms.” (lines 314–317).

2. *Trust as pivotal – add a brief link to the 3C model of vaccine hesitancy (confidence, complacency, convenience). Trust aligns with ‘confidence’.*

**Response:** We have added a brief mapping to the 3C model: “The salience of trust in our findings aligns closely with the ‘3C’ model of vaccine hesitancy (confidence, complacency and convenience), where the trust dimensions described here map most directly onto the ‘confidence’ component—that is, trust in the vaccine, in the system delivering it, and in the policymakers who decide on it—while geographic and indirect cost barriers (Subthemes 4.1 and 4.3) reflect the ‘convenience’ component.” (lines 328–332).

3. *Historical medical mistrust – temper causal language.*

**Response:** This has been done as described in our response to Major Concern 4 above (lines 334–342).

4. *“Western settings such as the United Kingdom grant children under 16 a degree of autonomy… through Gillick Competence” – this comparison is interesting but somewhat tangential. Consider shortening or moving to a footnote.*

**Response:** We have shortened the Gillick Competence comparison from a long descriptive sentence to a brief contrasting clause within the existing paragraph: “…a contrast that has been noted with Western settings such as the United Kingdom, where children under 16 may exercise a degree of autonomy over health decisions through the legal principle of Gillick Competence.” (lines 352–355). We chose to retain it in the main text as a brief contrast rather than move it to a footnote, given that PLOS ONE typographically does not commonly use footnotes.

5. *Strengths and Limitations – add one more limitation: social desirability bias. In FGDs, participants may have been reluctant to express vaccine refusal views in front of acceptors. This may explain the difficulty recruiting decliners and the small number of decliners who participated.*

**Response:** We have added a new paragraph to the Strengths and Limitations section addressing social desirability bias: “Third, social desirability bias is a recognised limitation of this study. In the FGD setting, particularly in the acceptor groups, participants may have been reluctant to express vaccine-hesitant or vaccine-refusing views in front of peers who had vaccinated their children, especially given the broader pro-vaccination climate associated with the Kwara State campaign. This dynamic may also help explain the difficulty in recruiting decliners and the relatively small number of declining caregivers who agreed to take part.” (lines 408–413).

6. *“The small number of decliners means their perspectives… may not represent the full spectrum” – add: “Future research should specifically oversample decliners using targeted recruitment strategies (e.g., through community leaders in areas with known lower coverage).”*

**Response:** We have added this sentence to the Limitations section: “Future research should specifically oversample decliners using targeted recruitment strategies (e.g., approaching community leaders in areas with documented lower coverage and conducting individual interviews where group settings may inhibit candour about refusal).” (lines 404–407). We have included individual interviews as an additional strategy in light of the social desirability point above.

**Minor Revisions – Implications**

1. *“The National Orientation Agency… could develop strategies to counter misinformation” – add that these strategies should be pre-tested with target communities to avoid unintended reinforcement of misinformation.*

**Response:** We have added the following: “Importantly, any such counter-misinformation messaging should be pre-tested with target communities (for example, through community advisory panels and small-scale message-testing focus groups) prior to large-scale rollout, in order to avoid unintentionally reinforcing the very rumours the messaging seeks to dispel.” (lines 419–423).

2. *“high quality research involving co-production with marginalised Fulani-Hausa communities” – add that this research should use qualitative methods conducted in Hausa or Fulfude by native speakers.*

**Response:** We have added: “such research should be conducted using qualitative methods in Hausa or Fulfulde by native speakers, ideally co-investigators or community researchers from the same communities, to ensure cultural and linguistic authenticity.” (lines 427–429).

3. *“house-to-house approach… successfully implemented during oral polio vaccination campaigns” – add that this approach is resource-intensive; discuss feasibility and cost implications.*

**Response:** We have added a feasibility/cost caveat: “We acknowledge, however, that house-to-house delivery is more resource-intensive than fixed-post or school-based delivery, with substantially higher requirements in terms of trained personnel, transport, cold-chain logistics and supervision. Any decision to scale up this approach should therefore be informed by an explicit assessment of cost, feasibility, and the incremental coverage gain achieved over less intensive delivery models.” (lines 434–438).

**Minor Revisions – Conclusion**

1. *Add a sentence on the need for quantitative studies to assess the prevalence of the barriers identified here (e.g., what proportion of caregivers endorse fertility concerns?).*

**Response:** We have added the following closing sentence to the Conclusion: “There is now a need for quantitative studies, both in Kwara State and other Nigerian states, to assess the prevalence of the specific barriers identified here—such as the proportion of caregivers endorsing fertility concerns, perceiving vaccinators as inadequately trained, or facing transport-related barriers—so that future interventions can be appropriately targeted.” (lines 446–450).

**Language and Presentation**

1. *“Before 2023, the high cost of HPV vaccines in Nigeria created significant access barriers” – change “created” to “posed.”*

**Response:** This has been amended (line 51).

2. *“Ilorin East local government area (LGA)” – capitalise “Local Government Area” for consistency.*

**Response:** This has been corrected throughout the manuscript, including the Abstract (line 20), the Introduction (line 70), and elsewhere.

3. *“Familiarization of the data was done through repeated reading” – change “was done” to “began with” for active voice.*

**Response:** This has been amended: “Familiarisation with the data began with repeated reading of transcripts…” (line 150).

4. *“quotes were selected to support themes and sub-themes” – add “illustrative” before “quotes.”*

**Response:** This has been amended: “Illustrative quotes were selected to support themes and sub-themes” (line 160).

5. *Subtheme 1.4 – the quote ends with “(R03, F, Urban, Decliner)” but R03 earlier was male. Check consistency. R03 appears multiple times with different genders.*

**Response:** Thank you for raising this. We have reviewed the apparent inconsistency and confirm that the labelling is in fact internally consistent with our coding convention. Participant identifiers in this study were assigned within each focus group rather than across the dataset as a whole, so "R03" denotes the third respondent in a given FGD, not a single individual across the study. Because we conducted five separate FGDs in different urban and rural locations and across both acceptor and decliner groups, the same numerical identifier (e.g. R03) legitimately refers to different participants in different groups, who differ in sex, residence, and vaccination decision. To make this transparent, every quotation throughout the Results is accompanied by the full set of contextual descriptors (participant number, sex, residence, acceptor/decliner status), so each quote remains uniquely and unambiguously attributable. We have re-checked all participant identifiers in the Results and confirm that each is internally consistent within its FGD and that no quotation has been misattributed. We have added a brief note to the Methods section to make this convention explicit for readers (Lines 161*–* 164).

6. *Tables and Figures – Table 1 verify age of child sums (currently 42, not 41); add Total rows; Table 2 add page numbers for where each theme is discussed.*

**Response:** Table 1 has been revised: Total rows have been added for Age, Gender, Age of child, Marital status, and Rural/urban; Gender percentages were also corrected (29.3% / 70.7%). In Table 2 (line 177) we have added page numbers in the main text where each subtheme is discussed [e.g., 1. Trust as a catalyst or barrier to HPV vaccination (Page 11)]

7. Appendix A (FGD guide): *The file is referenced but not visible in the PDF. Ensure it is included in the final submission*

**Response**: We thank the reviewer for noting this. The FGD guide is included as a Supporting Information file (S1 File: Focus Group Discussion Guide) with this revised submission, and the Methods section already references this file. We confirm we have uploaded it through the PLOS submission system and have added its citation to a Supporting Information section after the References.

8. References — formatting and missing details

**Response**: We thank the reviewer for the careful reference check. We have made the following amendments to the reference list:

Reference 2 (WHO Regional Office for Africa): added the access date "[accessed 29 May 2025]" to the URL.

Reference 8 (Agha & Nsofor): year confirmed as 2025 (published online 8 July 2025); DOI added as 10.1371/journal.pone.0325684.

Reference 21 (Gauna et al.): the journal name "Human Vaccines & Immunotherapeutics" has been added in full; previously this had been mistakenly truncated.

We have standardised the reference list throughout: every reference now includes (a) the journal/publisher full name, (b) volume and issue numbers where applicable, (c) page or article-locator numbers, (d) DOI where one exists, and (e) access date for any URL-only sources. References without a DOI are those (e.g. news pieces, agency reports) for which no DOI is assigned.

9. Ethics Statement — standalone section with approval numbers

**Response**: We have added the approval reference numbers to the Ethics Statement. Please refer to the third response in the methods section above.

We hope these revisions adequately address the reviewer’s concerns and that the manuscript is now suitable for publication in PLOS ONE. We are grateful again for the careful and constructive review.

Yours sincerely,

Abdulmujeeb Opeyemi Muhammad-Olodo, on behalf of all authors
