# Supplementary material for: Understanding factors influencing HPV vaccine uptake among caregivers in Kwara State, Nigeria: A qualitative study
Source: PLoS One. 2026 Jun 16;21(6):e0351659. doi: 10.1371/journal.pone.0351659 (PMC13271438; doi:10.1371/journal.pone.0351659)
Supplement: Peer review history

# Peer review: Decision Letter 0

**DOI:** 10.1371/journal.pone.0351659.r001

**Authors:** Morufu Raimi

4 May 2026

PONE-D-26-17339

Understanding factors influencing HPV vaccine uptake among caregivers in Kwara state, Nigeria: A qualitative study

PLOS One

Dear Dr. Asher,

Thank you for submitting your manuscript to PLOS ONE. After careful consideration, we feel that it has merit but does not fully meet PLOS ONE’s publication criteria as it currently stands. Therefore, we invite you to submit a revised version of the manuscript that addresses the points raised during the review process.

Please submit your revised manuscript by Jun 18 2026 11:59PM. If you will need more time than this to complete your revisions, please reply to this message or contact the journal office at plosone@plos.org. When you're ready to submit your revision, log on to https://www.editorialmanager.com/pone/ and select the 'Submissions Needing Revision' folder to locate your manuscript file.

Please include the following items when submitting your revised manuscript:

- A letter that responds to each point raised by the academic editor and reviewer(s). You should upload this letter as a separate file labeled 'Response to Reviewers'.
- A marked-up copy of your manuscript that highlights changes made to the original version. You should upload this as a separate file labeled 'Revised Manuscript with Track Changes'.
- An unmarked version of your revised paper without tracked changes. You should upload this as a separate file labeled 'Manuscript'.

If you would like to make changes to your financial disclosure, please include your updated statement in your cover letter. Guidelines for resubmitting your figure files are available below the reviewer comments at the end of this letter.

If applicable, we recommend that you deposit your laboratory protocols in protocols.io to enhance the reproducibility of your results. Protocols.io assigns your protocol its own identifier (DOI) so that it can be cited independently in the future. For instructions see: https://journals.plos.org/plosone/s/submission-guidelines#loc-laboratory-protocols. Additionally, PLOS ONE offers an option for publishing peer-reviewed Lab Protocol articles, which describe protocols hosted on protocols.io. Read more information on sharing protocols at https://plos.org/protocols?utm_medium=editorial-email&utm_source=authorletters&utm_campaign=protocols.

As the corresponding author, your ORCID iD is verified in the submission system and will appear in the published article. PLOS supports the use of ORCID, and we encourage all coauthors to register for an ORCID iD and use it as well. Please encourage your coauthors to verify their ORCID iD within the submission system before final acceptance, as unverified ORCID iDs will not appear in the published article. *Only*   the individual author can complete the verification step; PLOS staff *cannot*   verify ORCID iDs on behalf of authors.

We look forward to receiving your revised manuscript.

Kind regards,

Morufu Olalekan Raimi, Ph.D

Academic Editor

PLOS One

Journal Requirements:

When submitting your revision, we need you to address these additional requirements.

1. Please ensure that your manuscript meets PLOS ONE's style requirements, including those for file naming. The PLOS ONE style templates can be found at

https://journals.plos.org/plosone/s/file?id=wjVg/PLOSOne_formatting_sample_main_body.pdf and

https://journals.plos.org/plosone/s/file?id=ba62/PLOSOne_formatting_sample_title_authors_affiliations.pdf

2. Please include a complete copy of PLOS’ questionnaire on inclusivity in global research in your revised manuscript. Our policy for research in this area aims to improve transparency in the reporting of research performed outside of researchers’ own country or community. The policy applies to researchers who have travelled to a different country to conduct research, research with Indigenous populations or their lands, and research on cultural artefacts. The questionnaire can also be requested at the journal’s discretion for any other submissions, even if these conditions are not met.  Please find more information on the policy and a link to download a blank copy of the questionnaire here: https://journals.plos.org/plosone/s/best-practices-in-research-reporting. Please upload a completed version of your questionnaire as Supporting Information when you resubmit your manuscript.

3. Thank you for stating the following in the Acknowledgments Section of your manuscript:

“Abdulmujeeb O. Muhammad-Olodo reports financial support was provided by Commonwealth Scholarship Commission (CSC). The CSC had no other contribution to the report including study design; in the collection, analysis and interpretation of data; in the writing of the report; and in the decision to submit the article for publication.”

We note that you have provided funding information that is not currently declared in your Funding Statement. However, funding information should not appear in the Acknowledgments section or other areas of your manuscript. We will only publish funding information present in the Funding Statement section of the online submission form.

Please remove any funding-related text from the manuscript and let us know how you would like to update your Funding Statement. Currently, your Funding Statement reads as follows:

“Abdulmujeeb O. Muhammad-Olodo reports financial support was provided by Commonwealth Scholarship Commission (CSC). The CSC had no other contribution to the report including study design; in the collection, analysis and interpretation of data; in the writing of the report; and in the decision to submit the article for publication.”

Please include your amended statements within your cover letter; we will change the online submission form on your behalf.

4. Thank you for stating the following financial disclosure:

“Abdulmujeeb O. Muhammad-Olodo reports financial support was provided by Commonwealth Scholarship Commission (CSC). The CSC had no other contribution to the report including study design; in the collection, analysis and interpretation of data; in the writing of the report; and in the decision to submit the article for publication.”

Please state what role the funders took in the study.  If the funders had no role, please state: "The funders had no role in study design, data collection and analysis, decision to publish, or preparation of the manuscript."

If this statement is not correct you must amend it as needed.

Please include this amended Role of Funder statement in your cover letter; we will change the online submission form on your behalf.

5. In the online submission form you indicate that your data is not available for proprietary reasons and have provided a contact point for accessing this data. Please note that your current contact point is a co-author on this manuscript. According to our Data Policy, the contact point must not be an author on the manuscript and must be an institutional contact, ideally not an individual. Please revise your data statement to a non-author institutional point of contact, such as a data access or ethics committee, and send this to us via return email. Please also include contact information for the third party organization, and please include the full citation of where the data can be found.

6. If the reviewer comments include a recommendation to cite specific previously published works, please review and evaluate these publications to determine whether they are relevant and should be cited. There is no requirement to cite these works unless the editor has indicated otherwise.

7. Please review your reference list to ensure that it is complete and correct. If you have cited papers that have been retracted, please include the rationale for doing so in the manuscript text, or remove these references and replace them with relevant current references. Any changes to the reference list should be mentioned in the rebuttal letter that accompanies your revised manuscript. If you need to cite a retracted article, indicate the article’s retracted status in the References list and also include a citation and full reference for the retraction notice.

Additional Editor Comments:

Manuscript Number: PONE-D-26-17339

Title: Understanding factors influencing HPV vaccine uptake among caregivers in Kwara state, Nigeria: A qualitative study

Recommendation: Minor Revision

Reviewer: Morufu Olalekan Raimi, PhD (Chief Editor, PLOS One – Vaccine Acceptance & Global Health)

Review Date: 2026-04-30

Reviewer Comments

Dear Authors,

Thank you for submitting this qualitative study on HPV vaccine uptake among caregivers in Kwara State, Nigeria. The manuscript addresses a critically important public health issue: why HPV vaccination coverage remains suboptimal in Nigeria despite free provision. The qualitative approach is appropriate for exploring the complex, context-specific factors influencing caregiver decisions, and the findings, particularly around multi-level trust, historical medical mistrust, and implementation gaps, offer valuable insights for policy and programme improvement. The manuscript is well-written, methodologically sound in most respects, and makes a meaningful contribution to the literature on vaccine acceptance in low- and middle-income settings. However, several issues require attention before the manuscript meets PLOS ONE standards. These are moderate in scope and focus on data availability, methodological clarity, interpretation of findings, and presentation.

Recommendation: Minor Revision – The manuscript is suitable for publication after addressing the issues below.

Major Concerns

1. Data Availability – Non-Compliant with PLOS ONE Policy

The Data Availability Statement (page 7) states: “Participants in this study did not consent to the public sharing of their raw transcripts. Due to the sensitive nature of the qualitative interviews and the risk of re-identification, data are available upon request from the corresponding author for researchers who meet the criteria for access to confidential data.”

While this is a common approach for qualitative data, PLOS ONE requires that data underlying the findings be fully available without restriction or a clear explanation of why restrictions are necessary. The current statement is vague (“researchers who meet the criteria” – what criteria?) and does not specify who will review requests or on what basis they might be denied.

Required action:

• Specify the exact conditions under which data will be shared (e.g., “Data will be shared with researchers who provide a methodologically sound research proposal and sign a data access agreement prohibiting re-identification and onward sharing.”)

• OR, if ethical approval explicitly prohibits data sharing, state this clearly and provide the relevant ethics committee approval letter as supporting information.

• Additionally, provide anonymized transcripts (with all identifiable information removed) as supporting information if possible. If not possible, explain why.

Acceptable revision:

“Raw transcripts cannot be shared publicly because participants did not consent to public data sharing. Anonymized transcripts (with names, locations, and other identifiers removed) are available from the corresponding author for researchers who submit a methodologically sound research proposal and sign a data access agreement. Requests will be reviewed by the University of Nottingham Faculty of Medicine and Health Sciences Research Ethics Committee.”

2. Limited Representation of Vaccine Decliners – Potential Bias

The study includes 35 acceptors (five FGDs? Actually: four FGDs with acceptors, n=35) and only 6 decliners (one FGD) . The manuscript acknowledges this as a limitation (lines 383–389), stating that recruitment of decliners was challenging given intensive vaccination efforts.

However, the analysis and discussion treat the findings as broadly representative of factors influencing both acceptance and declination. With such a small and potentially unrepresentative sample of decliners, there is a risk that:

• Factors specific to decliners may be under-identified.

• The single decliner FGD (urban) may not capture rural decliner perspectives.

• Thematic saturation for decliner perspectives was almost certainly not achieved.

Required action:

• Reframe the manuscript to more clearly emphasize that the findings primarily reflect acceptor perspectives, with decliner insights offered as exploratory.

• In the Discussion, add a sentence stating: *“Given the small number of decliners (n=6), our findings on barriers and reasons for refusal should be considered preliminary and require further investigation in larger samples of vaccine-declining caregivers.”*

• Consider renaming the study to reflect the acceptor focus (e.g., “Factors influencing HPV vaccine uptake and non-uptake…” but with emphasis on acceptors).

3. Missing Information on Recruitment and Sampling

The Methods section (lines 115-120) describes recruitment via community mobilizers and WhatsApp groups. However, several details are missing:

• How were community mobilizers selected? Were they health workers, community leaders, or lay volunteers?

• What was the response rate? How many caregivers were approached? How many declined to participate? This is important for assessing potential selection bias (e.g., acceptors may have been more willing to participate).

• How was saturation determined? The manuscript mentions “Data saturation was monitored during coding” (line 142) but does not specify how many FGDs were conducted before saturation was reached, or whether any additional FGDs were planned but not conducted.

Required action:

• Add a paragraph in Methods describing the recruitment process in more detail, including the roles of community mobilizers, the number of caregivers approached, and the number who declined.

• Specify how saturation was assessed (e.g., “Saturation was considered reached when three consecutive FGDs yielded no new codes.”).

4. Overstatement of Findings on “Historical Medical Mistrust”

The manuscript makes strong claims about historical medical exploitation (e.g., lines 319–326: “The legacy of unethical medical experimentation has created lasting mistrust… from German-backed sterilization tests… to the Pfizer meningitis drug trial”). While these historical events are documented, the manuscript presents them as directly causative of current HPV vaccine hesitancy without empirical evidence from the participants themselves.

The participant quotes (lines 203-215) mention “rumours that the whites are trying to use it to reduce our population” and COVID-19 vaccine experiences, but no participant explicitly mentioned the Pfizer trial or colonial-era medical experiments. The connection is inferential, not directly reported.

Required action:

• Temper the language to reflect that these historical events may contribute to a broader context of mistrust, rather than stating they directly cause current hesitancy.

• Change “explain why population control narratives resonate so powerfully as they echo real experiences” to “may help explain why population control narratives resonate, as they echo documented historical experiences of medical exploitation.”

• Ensure that the manuscript does not attribute to participants beliefs or knowledge they did not explicitly express.

Minor Revisions

Abstract

• Line 28: “Despite removing cost barriers, misinformation about fertility impacts and population control resulted in variable uptake across states” – change “resulted in” to “contributed to” (causality cannot be established from this study alone).

• Line 36: “Four themes emerged from analysis” – add “(Table 2)” for clarity.

• Line 42: “free vaccine provision alone is insufficient” – good. Add “but not sufficient” after “free provision.”

Introduction

• Line 50-52: “Cervical cancer is the fourth leading cause of death amongst women globally, with more than 342,000 deaths in 2020” – provide reference (Sung et al., 2021 – already in references, line 418). Good.

• Line 60-62: “the Federal Government of Nigeria… partnered with… Gavi, IFFIm, and WHO” – add the year (2023) earlier in the sentence for clarity.

• Line 72-75: “Understanding what influenced caregivers… is crucial to understand this low response rate” – change second “understand” to “addressing.”

Methods

• Line 84-86: “This study was underpinned by the constructionist paradigm” – good. Add a sentence on how this paradigm influenced data collection and analysis (e.g., “We therefore focused on how shared social meanings, rather than individual psychological traits, shaped vaccination decisions.”)

• Line 89-91: “Focus group discussions (FGDs) were considered most appropriate” – justify why FGDs rather than individual interviews. Mention that FGDs allow for interaction and identification of shared community norms.

• Line 95-97: Ethical approval – provide approval numbers for both committees (UoN and UITH). Currently missing.

• Line 108–110: “The study population comprised 41 caregivers” – add the total number of eligible children represented (if known).

• Line 115-121: Recruitment – as noted above, add more detail on response rate and mobilizer roles.

• Line 124-126: “We held four FGDs with accepting caregivers… and one FGD with declining caregivers” – specify that the decliner FGD was urban only. Add: “We were unable to recruit decliners from rural areas.”

• Line 135-136: “Participants received 2,500 (equivalent to £5) as compensation” – specify the currency (2,500 Nigerian Naira). Add that this amount was ethically approved and did not constitute undue inducement.

Results

• Table 1 (page 18–19): The table is clear. Add a footnote: “Percentages may not sum to 100 due to rounding.” Also, “Age of child” – add total n? It sums to 41? Check: 4+8+3+3+11+13 = 42. Correct to 41.

• Theme 1 (page 20-22): Well presented. Add a quote from a Fulani-Hausa participant if available (acknowledged that none were directly included – see limitation).

• Subtheme 1.2 (line 187-191): The quote about inadequately trained personnel (R03, F, Urban, Decliner) is important. Add a brief interpretive comment linking this to trust in health system competence.

• Subtheme 1.4 (line 203-215): The claim about COVID-19 experiences is supported by a quote (line 209–210). However, the quote does not explicitly mention COVID-19 – it says “she did not take the vaccine [during COVID-19?]” – clarify. The participant says “during COVID… because of the rumour” – acceptable but ensure the quote is fully presented.

• Subtheme 3.1 (line 232-239): “inadequate pre-vaccination counselling about potential side effects deterred others” – add a quote that explicitly mentions lack of counselling. The existing quote (R04, M, Urban, Decliner) says “they did not explain the implications, the side effect” – acceptable.

• Subtheme 4.1 (line 250-262): School-based delivery gaps – excellent point. Add a recommendation from participants about how to improve this (e.g., advance notice, letters sent home with students).

Discussion

• Line 294-297: “Radio emerged as the primary mass media source” – good. Add that radio reach in rural areas is often higher than internet/social media, which is relevant for intervention design.

• Line 306-313: Trust as pivotal – excellent. Add a brief link to the 3C model of vaccine hesitancy (confidence, complacency, convenience) – trust aligns with “confidence.”

• Line 319–326: Historical medical mistrust – as noted above, temper causal language.

• Line 336-338: “Western settings such as the United Kingdom grant children under 16 a degree of autonomy… through Gillick Competence” – this is an interesting comparison but somewhat tangential. Consider shortening or moving to a footnote.

• Line 367-373: “Strengths and Limitations” – add one more limitation: social desirability bias. In FGDs, participants may have been reluctant to express vaccine refusal views in front of acceptors. This may explain the difficulty recruiting decliners and the small number of decliners who participated.

• Line 385-389: “The small number of decliners means their perspectives… may not represent the full spectrum” – good. Add: “Future research should specifically oversample decliners using targeted recruitment strategies (e.g., through community leaders in areas with known lower coverage).”

Implications (lines 390-410)

• Line 393-396: “The National Orientation Agency… could develop strategies to counter misinformation” – specific and actionable. Add that these strategies should be pre-tested with target communities to avoid unintended reinforcement of misinformation.

• Line 398-400: “high quality research involving co-production with marginalised Fulani-Hausa communities” – excellent. Add that this research should use qualitative methods conducted in Hausa or Fulfude by native speakers.

• Line 402-406: “house-to-house approach… successfully implemented during oral polio vaccination campaigns” – good. Add that this approach is resource-intensive; discuss feasibility and cost implications.

Conclusion

• Line 408-414: “This study provides insights into HPV vaccination experiences in Nigeria” – good. Add a sentence on the need for quantitative studies to assess the prevalence of the barriers identified here (e.g., what proportion of caregivers endorse fertility concerns?).

Language and Presentation

Grammar and Style

• Line 56: “Before 2023, the high cost of HPV vaccines in Nigeria created significant access barriers” – change “created” to “posed.”

• Line 77: “Ilorin East local government area (LGA)” – capitalize “Local Government Area” for consistency.

• Line 143-144: “Familiarization of the data was done through repeated reading” – change “was done” to “began with” for active voice.

• Line 150-151: “quotes were selected to support themes and sub-themes” – add “illustrative” before “quotes.”

• Line 203-215: The quote in Subtheme 1.4 ends with “(R03, F, Urban, Decliner)” – but R03 earlier was male (line 182). Check consistency. R03 appears multiple times with different genders. Correct.

Tables and Figures

• Table 1 (page 18-19): As noted, verify age of child frequencies (sum to 42, not 41). Also, “Marital status” – add “Total” row.

• Table 2 (page 19-20): Well organized. Add page numbers for where each theme is discussed.

• Appendix A (FGD guide): The file is referenced but not visible in the PDF. Ensure it is included in the final submission.

References

• Reference 1 (Sung et al.) – correct.

• Reference 2 (WHO Regional Office for Africa) – URL provided. Add access date.

• Reference 8 (Agha & Nsofor, 2025) – PLOS One. Verify year (2025 is future relative to submission? Acceptable if published online ahead of print).

• Reference 21 (Gauna et al.) – missing journal name? It is “Human Vaccines & Immunotherapeutics.” Add.

• Reference 31 (Richards et al.) – correct.

• Inconsistent formatting: Some references have DOIs, others do not. Add DOIs where available. Some have issue numbers, others do not. Standardize.

Ethics Statement

• The ethics statement on page 4 is clear. However, the approval numbers are missing. Add: “University of Nottingham Faculty of Medicine and Health Sciences Research Ethics Committee (approval number: [XXX]) and University of Ilorin Teaching Hospital Research Ethics Committee (approval number: [XXX]).”

Data Availability – Additional Note

The manuscript states (page 8) that “data are available upon request from the corresponding author.” As noted above, this is insufficient for PLOS ONE. In addition to the revisions suggested, please ensure that the Data Availability Statement in the manuscript matches the information provided in the submission system. Currently, the submission system states “No – some restrictions will apply” (page 6) while the manuscript text says data are available upon request. These must be consistent.

Decision Rationale

This qualitative study provides valuable, context-specific insights into HPV vaccine decision-making among caregivers in Kwara State, Nigeria. The findings on multi-level trust, historical medical mistrust, personal cancer experiences, and implementation gaps are policy-relevant and contribute to the growing literature on vaccine acceptance in Africa. The manuscript is well-written and methodologically sound in most respects.

However, the manuscript cannot be accepted in its current form due to:

1. Non-compliance with PLOS ONE data availability policy (data available “upon request” without clear conditions; no anonymized transcripts provided).

2. Limited representation of vaccine decliners (n=6, one FGD) leading to potential bias in findings on barriers to uptake.

3. Missing methodological details (recruitment response rate, saturation criteria, ethics approval numbers).

4. Overstatement of historical mistrust claims without direct participant quotations.

These issues are fixable with moderate revisions. The authors do not need to collect new data, but they must clarify the data availability conditions, temper the historical mistrust interpretation, and provide missing methodological details.

Recommendation: Minor Revision – I look forward to seeing the revised manuscript.

Sincerely,

Morufu Olalekan Raimi, PhD

Academic Editor (Environmental Epidemiology)

PLOS One

[Note: HTML markup is below. Please do not edit.]

Reviewers' comments:

Reviewer's Responses to Questions

**Comments to the Author**

1. Is the manuscript technically sound, and do the data support the conclusions?

The manuscript must describe a technically sound piece of scientific research with data that supports the conclusions. Experiments must have been conducted rigorously, with appropriate controls, replication, and sample sizes. The conclusions must be drawn appropriately based on the data presented.

Reviewer #1: Yes

**********

2. Has the statistical analysis been performed appropriately and rigorously?

Reviewer #1: Yes

**********

3. Have the authors made all data underlying the findings in their manuscript fully available?

The PLOS Data policy requires authors to make all data underlying the findings described in their manuscript fully available without restriction, with rare exception (please refer to the Data Availability Statement in the manuscript PDF file). The data should be provided as part of the manuscript or its supporting information, or deposited to a public repository. For example, in addition to summary statistics, the data points behind means, medians and variance measures should be available. If there are restrictions on publicly sharing data—e.g. participant privacy or use of data from a third party—those must be specified.

Reviewer #1: Yes

**********

4. Is the manuscript presented in an intelligible fashion and written in standard English?

PLOS ONE does not copyedit accepted manuscripts, so the language in submitted articles must be clear, correct, and unambiguous. Any typographical or grammatical errors should be corrected at revision, so please note any specific errors here.

Reviewer #1: Yes

**********

5. Review Comments to the Author

Please use the space provided to explain your answers to the questions above. You may also include additional comments for the author, including concerns about dual publication, research ethics, or publication ethics. (Please upload your review as an attachment if it exceeds 20,000 characters)

Reviewer #1: This is a highly relevant and insightful qualitative study. Understanding the barriers and facilitators to HPV vaccine uptake in Nigeria, especially following the recent 2023 campaign, is crucial for improving global cervical cancer prevention.

I appreciate your rigorous use of reflexive thematic analysis and the deliberate inclusion of both vaccine acceptors and decliners in your focus groups. Your findings regarding the multi-layered nature of trust, the impact of historical medical mistrust, and the specific geographic and language barriers facing certain communities provide highly actionable insights for future public health campaigns.

I have no changes to request. Congratulations on a well-executed and valuable paper.

**********

6. PLOS authors have the option to publish the peer review history of their article (what does this mean?). If published, this will include your full peer review and any attached files.

If you choose “no”, your identity will remain anonymous but your review may still be made public.

**Do you want your identity to be public for this peer review?**   For information about this choice, including consent withdrawal, please see our Privacy Policy.

Reviewer #1: **Yes:**  Ganesh Praneeth Roy Avula

**********

[NOTE: If reviewer comments were submitted as an attachment file, they will be attached to this email and accessible via the submission site. Please log into your account, locate the manuscript record, and check for the action link "View Attachments". If this link does not appear, there are no attachment files.]

To ensure your figures meet our technical requirements, please review our figure guidelines: https://journals.plos.org/plosone/s/figures

You may also use PLOS’s free figure tool, NAAS, to help you prepare publication quality figures: https://journals.plos.org/plosone/s/figures#loc-tools-for-figure-preparation.

NAAS will assess whether your figures meet our technical requirements by comparing each figure against our figure specifications.

# Author comment: Author response to Decision Letter 1

**DOI:** 10.1371/journal.pone.0351659.r002

22 May 2026

We are grateful to the reviewer for their thorough and constructive feedback on our manuscript. The comments have helped us substantially strengthen the methodological clarity, balance the framing of our findings, and refine the language and presentation of the paper. We have addressed every point raised below, indicating the relevant line numbers in the revised manuscript where changes have been made. Our amendments and responses are detailed below.

Major Concerns

1. Data Availability Statement

Response: We thank the reviewer and the editor for highlighting this. We agree that our previous Data Availability Statement was insufficiently specific and we have revised it to comply with PLOS ONE policy and to align the manuscript text with the submission system. Because the qualitative material carries a residual risk of re-identification (small community, distinctive personal narratives), unrestricted public deposition is not appropriate. Anonymised transcripts are however available to qualified researchers upon reasonable request, with access governed by the institutional ethics committee that approved this study. The revised Data Availability Statement now reads:

"The qualitative data generated during this study contain potentially identifying information and cannot be made publicly available, given the residual risk of re-identification arising from the small community in which the study was conducted and the distinctive personal narratives shared by participants. Anonymised transcripts and the FGD guide may be made available to qualified researchers who submit a methodologically sound research proposal and sign a data access agreement prohibiting re-identification and onward sharing. Requests should be directed to the University of Nottingham Faculty of Medicine and Health Sciences Research Ethics Committee (Reference: FMHS 112-0325), which can be contacted at [fmhs-researchethics@nottingham.ac.uk]. The committee, not the authors, will determine whether each request meets the conditions of the original ethical approval."

We have also updated the Data Availability Statement in the submission system so that it matches this manuscript text exactly, and we have changed the system selection to the PLOS option that most closely corresponds to "data are restricted by ethics and available on request through a non-author contact."

2. Limited Representation of Vaccine Decliners – Potential Bias. The study includes 35 acceptors (four FGDs) and only 6 decliners (one FGD). The manuscript acknowledges this as a limitation but the analysis and discussion treat the findings as broadly representative of factors influencing both acceptance and declination. With such a small and potentially unrepresentative sample of decliners, factors specific to decliners may be under-identified, the single (urban) decliner FGD may not capture rural decliner perspectives, and thematic saturation for decliner perspectives was almost certainly not achieved. The reviewer requested that the manuscript be reframed to emphasise that the findings primarily reflect acceptor perspectives, that a sentence be added in the Discussion stating the decliner findings should be considered preliminary, and that we consider how the title reflects this.

Response: We thank the reviewer for this important point and agree that the framing required adjustment. We have made the following changes:

• In the Strengths and Limitations section, we have added the sentence: “Given the small number of decliners (n=6), our findings on barriers and reasons for refusal should be considered preliminary and require further investigation in larger samples of vaccine-declining caregivers. Thematic saturation for decliner perspectives was almost certainly not achieved, and the single decliner FGD was conducted in an urban setting, so rural decliner perspectives are not represented in our data. The analysis and conclusions in this study therefore primarily reflect acceptor perspectives, with decliner insights presented as exploratory.” (lines 400–404 of the revised manuscript).

• We have also added a sentence on targeted future recruitment strategies for decliners (lines 405–408).

• In the Methods (Data Collection) section we have explicitly stated that “the decliner FGD was conducted in an urban setting only, as we were unable to recruit a sufficient number of decliners from rural areas to form a separate group” (lines 131–132).

After careful consideration, we have decided not to alter the title of the paper. The current title (“Understanding factors influencing HPV vaccine uptake…”) refers to uptake decisions in general, which encompass both acceptance and refusal, and we feel this remains accurate. We believe the explicit reframing in the Limitations and Discussion sections, combined with the new clarifying language at the start of the relevant Methods passages, adequately signals the acceptor focus of the analysis without changing the title.

3. Missing Information on Recruitment and Sampling. Several details are missing: how community mobilizers were selected (health workers, community leaders, lay volunteers); the response rate (how many caregivers were approached, how many declined); and how saturation was determined. Please add a paragraph in Methods describing the recruitment process in more detail and specify how saturation was assessed.

Response: We have substantially expanded the recruitment description in the Methods section. The revised paragraph (lines 112–124) now reads: “The community mobilizers were lay community members nominated by local community leaders (Baales and ward heads) for their familiarity with residents and their existing relationships within places of worship and markets; they were not health workers and received a brief verbal orientation about the study’s purpose, eligibility criteria and the importance of recruiting both acceptors and decliners. Mobilizers helped identify caregivers known to have either accepted or declined vaccination during the campaign and made initial introductions, after which the research assistant explained the study and confirmed eligibility. Approximately 60 caregivers were approached across the five communities. Of these, 41 agreed to participate (response rate ≈ 68%); the remainder either could not attend the scheduled session due to work or domestic commitments, or declined to take part without giving a specific reason. Recruitment of acceptors was relatively straightforward, whereas decliners were more difficult to identify and persuade to attend, which we revisit in the Strengths and Limitations section.”

In the Data analysis section we have also clarified how saturation was assessed: “Saturation was assessed during analysis and was considered reached when three consecutive FGDs yielded no new codes; on this basis no additional acceptor FGDs were conducted, although we acknowledge that saturation for decliner perspectives was almost certainly not achieved given that only one decliner FGD was conducted (see Strengths and Limitations).” (lines 152–156).

4. Overstatement of Findings on “Historical Medical Mistrust”. The manuscript presents historical medical exploitation events (German-backed sterilisation tests, the Pfizer meningitis trial) as directly causative of current HPV vaccine hesitancy without empirical evidence from the participants themselves. No participant explicitly mentioned the Pfizer trial or colonial-era experiments. Please temper the language to reflect that these historical events may contribute to a broader context of mistrust, rather than stating they directly cause current hesitancy. Specifically change “explain why population control narratives resonate so powerfully as they echo real experiences” to “may help explain why population control narratives resonate, as they echo documented historical experiences of medical exploitation.” Ensure that the manuscript does not attribute to participants beliefs or knowledge they did not explicitly express.

Response: We thank the reviewer for this important methodological point and agree that the original wording overstated the causal link. We have rewritten the relevant Discussion paragraph (lines 335–347) to make clear that this is an inferential interpretation rather than a participant-reported attribution. The revised text now reads: “Conversely, historical medical mistrust appeared to manifest through fears that the vaccine was intended to control population levels through reducing fertility, echoing documented concerns about Western medical interventions in African contexts. A broader context of unethical medical experimentation has been documented in the literature—ranging from German-backed sterilisation tests on Herero women in the 1900s to the Pfizer meningitis drug trial in Kano, northern Nigeria, in which eleven children died during testing of an experimental antibiotic in the 1990s. Although none of our participants explicitly named these specific historical events, the recurrence of population-control narratives in our data may help explain why such narratives resonate, as they echo documented historical experiences of medical exploitation in the region. The COVID-19 pandemic appeared to reactivate such concerns, as one decliner explicitly linked her current refusal to rumours encountered during the COVID-19 vaccination roll-out, suggesting that perceived adverse experiences with one vaccination programme may contribute to hesitancy towards subsequent ones.”

We have also softened the related abstract sentence (“may have manifested as fertility and population control fears”, line 31) and the introductory Discussion paragraph (“appeared to create substantial barriers”, line 303), so that all causal claims about historical mistrust are now appropriately hedged.

Minor Revisions – Abstract

1. “resulted in variable uptake across states” – change “resulted in” to “contributed to” (causality cannot be established from this study alone).

Response: We have made this change. The Abstract now reads “…misinformation about fertility impacts and population control contributed to variable uptake across states” (lines 17–18).

2. “Four themes emerged from analysis” – add “(Table 2)” for clarity.

Response: We have added the table reference: “Four themes emerged from analysis (Table 2)” (line 28).

3. “free vaccine provision alone is insufficient” – add “but not sufficient” after “free provision.”

Response: We have rephrased the conclusion sentence to: “Free vaccine provision is necessary but not sufficient.” (line 38).

Minor Revisions – Introduction

1. “Cervical cancer is the fourth leading cause of death amongst women globally…” – reference confirmed (Sung et al., 2021).

Response: Acknowledged. The reference is retained (line 46).

2. “the Federal Government of Nigeria… partnered with Gavi, IFFIm, and WHO” – add the year (2023) earlier in the sentence for clarity.

Response: We have moved the year to the beginning of the sentence: “To address this inequity, in October 2023 the Federal Government of Nigeria, through the Ministry of Health and National Primary Health Care Development Agency (NPHCDA), partnered with multilateral organisations…” (lines 54–56).

3. “Understanding what influenced caregivers… is crucial to understand this low response rate” – change second “understand” to “addressing.”

Response: This has been amended: “…is crucial to addressing this low response rate in order to improve future vaccination campaigns in Nigeria” (line 66).

Minor Revisions – Methods

1. “This study was underpinned by the constructionist paradigm” – add a sentence on how this paradigm influenced data collection and analysis.

Response: We have added the following sentence to clarify how the paradigm shaped our approach: “We therefore focused on how shared social meanings, rather than individual psychological traits, shaped vaccination decisions, allowing us to gain understanding of participants’ experiences and the thought-process behind the decisions they took.” (lines 83–85).

2. “Focus group discussions (FGDs) were considered most appropriate” – justify why FGDs rather than individual interviews. Mention that FGDs allow for interaction and identification of shared community norms.

Response: We have expanded this justification: “Focus group discussions (FGDs) were considered most appropriate for gaining rich insights into the community setting because they allow for interaction between participants and facilitate identification of shared community norms and group-level meanings, in addition to revealing diverse perspectives within the dynamics of the group.” (lines 85–89).

3. Ethical approval – provide approval numbers for both committees (UoN and UITH). Currently missing.

Response: We have added both approval reference numbers: “…granted by the University of Nottingham Faculty of Medicine and Health Sciences Research Ethics Committee (Reference: FMHS 112-0325) and the University of Ilorin Teaching Hospital (UITH) Research Ethics Committee (Reference: NHREC/02/05/2010).” (lines 90–93). The reference numbers are taken from the original ethics approval letters.

4. Recruitment – add more detail on response rate and mobilizer roles.

Response: This has been substantially expanded – see our response to Major Concern 3 above. The revised text now describes the lay status and orientation of mobilizers, gives the approximate number of caregivers approached (~60), and reports the response rate (≈68%) (lines 111–121).

5. “We held four FGDs with accepting caregivers… and one FGD with declining caregivers” – specify that the decliner FGD was urban only. Add: “We were unable to recruit decliners from rural areas.”

Response: We have made this explicit: “…the decliner FGD was conducted in an urban setting only, as we were unable to recruit a sufficient number of decliners from rural areas to form a separate group.” (lines 131–132).

6. “Participants received 2,500 (equivalent to £5) as compensation” – specify the currency (2,500 Nigerian Naira). Add that this amount was ethically approved and did not constitute undue inducement.

Response: We have specified the currency and added the ethical justification: “Participants received ₦2,500 Nigerian Naira (equivalent to approximately £5 at the time of the study) as compensation for their time and transportation costs. This amount was reviewed and approved by the ethics committees as appropriate compensation that did not constitute undue inducement, given local transport costs and the typical 60–90 minute time commitment required of participants.” (lines 141–145).

Minor Revisions – Results

1. Table 1 – add a footnote: “Percentages may not sum to 100 due to rounding.” Verify the “Age of child” frequencies, which sum to 42 not 41.

Response: The corrected "Age of child" frequencies now sum to 41, which is consistent with the total caregiver sample size, and the percentages have been recalculated accordingly. We have also added Total rows for each categorical variable as requested, and added the footnote "Percentages may not sum to 100 due to rounding."

2. Marital status – add a Total row.

Response: We have added Total rows to all categorical variables in Table 1, including Age, Gender, Age of child, Marital status, and Rural/urban. The percentages have also been recalculated where necessary; the Gender row now correctly reads 12 male (29.3%) and 29 female (70.7%) of the 41 participants.

3. Theme 1, Subtheme 1.2 – the quote about inadequately trained personnel (R03, F, Urban, Decliner) is important. Add a brief interpretive comment linking this to trust in health system competence.

Response: We have added the following interpretive sentence after the quote: “This account illustrates how perceived competence of vaccinators served as a proximal cue for the broader trustworthiness of the health system: when caregivers felt their questions were not adequately answered, doubts about vaccinator training appeared to generalise to doubts about the vaccine itself and the institutions delivering it.” (lines 202–205).

4. Subtheme 1.4 – the quote about COVID-19 experiences does not explicitly mention COVID-19. Clarify and ensure the quote is fully presented.

Response: We have replaced the previous summary sentence with a fully presented decliner quote that

Attachment Submitted filename: Response_to_Reviewers_PLoS_One_LA.docx

# Editor report: Decision Letter 1

**DOI:** 10.1371/journal.pone.0351659.r003

**Authors:** Morufu Raimi

31 May 2026

Understanding factors influencing HPV vaccine uptake among caregivers in Kwara state, Nigeria: A qualitative study

PONE-D-26-17339R1

Dear Author,

We’re pleased to inform you that your manuscript has been judged scientifically suitable for publication and will be formally accepted for publication once it meets all outstanding technical requirements.

Within one week, you’ll receive an e-mail detailing the required amendments. When these have been addressed, you’ll receive a formal acceptance letter and your manuscript will be scheduled for publication.

An invoice will be generated when your article is formally accepted. Please note, if your institution has a publishing partnership with PLOS and your article meets the relevant criteria, all or part of your publication costs will be covered. Please make sure your user information is up-to-date by logging into Editorial Manager at Editorial Manager® and clicking the ‘Update My Information' link at the top of the page. For questions related to billing, please contact billing support.

If your institution or institutions have a press office, please notify them about your upcoming paper to help maximize its impact. If they’ll be preparing press materials, please inform our press team as soon as possible -- no later than 48 hours after receiving the formal acceptance. Your manuscript will remain under strict press embargo until 2 pm Eastern Time on the date of publication. For more information, please contact onepress@plos.org.

Kind regards,

Morufu Olalekan Raimi, Ph.D

Academic Editor

PLOS One

Additional Editor Comments (optional):

PLOS ONE Editorial Decision

Manuscript ID: PONE-D-26-17339_R1

Title: Understanding factors influencing HPV vaccine uptake among caregivers in Kwara state, Nigeria: A qualitative study

Authors: Laura Asher, Abdulmujeeb O. Muhammad-Olodo

Editor: Dr. Morufu Olalekan Raimi, PhD (Academic Editor, PLOS ONE – Vaccine Acceptance & Global Health)

Date of Decision: 29 May 2026

Decision: Accept

Summary of Evaluation

The authors have submitted a thoroughly revised manuscript that addresses all major and minor concerns raised during the original review. The revisions are comprehensive, methodologically appropriate, and responsive to each point of critique. No new data collection or analytical reworking is required. The manuscript now meets PLOS ONE’s standards for publication in terms of scientific rigor, ethical transparency, and reporting quality.

Detailed Assessment of Revisions Against Prior Concerns

1. Data Availability (Previously Major Concern)

Resolved.

The revised Data Availability Statement is specific, actionable, and compliant with PLOS ONE policy. It now identifies the institutional ethics committee (University of Nottingham) as the gatekeeper, specifies the conditions for access (methodologically sound proposal + data access agreement), and explains the residual re-identification risk. The submission system entry has been aligned with the manuscript text.

2. Limited Representation of Vaccine Decliners (Previously Major Concern)

Resolved.

The authors have appropriately reframed the study’s findings as primarily reflecting acceptor perspectives, with decliner insights explicitly labelled exploratory. The Strengths and Limitations section now contains clear preliminary statements, acknowledges lack of saturation and rural decliner representation, and proposes targeted oversampling strategies. The title remains accurate and is not misleading given the revised framing.

3. Missing Recruitment & Sampling Details (Previously Major Concern)

Resolved.

The Methods section now includes: selection criteria for lay community mobilizers, approximate number approached (~60), response rate (~68%), and explicit saturation criteria (three consecutive FGDs with no new codes). All missing methodological information has been supplied.

4. Overstatement of Historical Medical Mistrust (Previously Major Concern)

Resolved.

The Discussion has been rewritten to temper causal language. The text now correctly frames historical events as providing a contextual backdrop that may help explain why population-control narratives resonate, rather than claiming direct causation. No participant is attributed with knowledge they did not express. Abstract and introductory Discussion sentences have also been appropriately hedged.

5. Minor Revisions (Abstract, Introduction, Methods, Results, Discussion, Implications, Conclusion, Language, Tables, References, Ethics Statement)

Resolved.

Every minor revision requested has been addressed systematically, including:

• Abstract

• Constructionist paradigm justification and FGD rationale

• Ethics approval numbers added

• Currency specification and compensation ethical justification

• Table 1 corrections (age of child sum, total rows, footnote)

• Interpretive comment linking vaccinator competence to system trust

• Fully presented COVID-19 quote

• Participant recommendation on school advance notices

• Radio reach in rural areas

• 3C model linkage

• Shortened Gillick Competence reference

• Social desirability bias added as limitation

• Counter-misinformation pre-testing, native-language research, and house-to-house cost feasibility added

• Quantitative study need in Conclusion

• Language corrections (posed, Local Government Area, began with, illustrative quotes)

• Participant identifier convention clarified

• Table 2 page numbers added

• Supporting Information (FGD guide) confirmed included

• Reference formatting standardised

• Ethics statement with approval numbers

Remaining Minor Copyediting Recommendations (Not Required for Acceptance)

The following are optional suggestions that do not affect scientific integrity or publication readiness. The journal’s production team may address these during typesetting:

1. Line 141: “£2,500 Nigerian Naira” – correct to “₦2,500” (the pound sign appears to be a typographical error; the text correctly uses “₦” elsewhere).

2. Line 177 (Table 2): Page numbers in parentheses – ensure they match the final pagination after typesetting (production will handle this).

3. Reference 21: Journal name is correct (“Human Vaccines & Immunotherapeutics”) – no further action needed.

4. Consistency in abbreviation: “UITh” vs “UITH” – the manuscript uses “UITH” in the ethics statement (line 91) – standardise to “UITH” throughout.

These do not require author revision at this stage.

Final Editorial Comment

This is a well-executed qualitative study that makes a meaningful contribution to understanding HPV vaccine decision-making in a real-world campaign context in Nigeria. The authors have demonstrated exemplary responsiveness to peer review, and the manuscript is now clear, balanced, and transparent about its methodological limitations. The findings have clear policy implications for Nigeria’s National Programme on Immunisation and similar LMIC settings.

Accept as is. Production will handle minor copyediting.

Sincerely,

Dr. Morufu Olalekan Raimi, PhD

Academic Editor (Environmental Epidemiology)

PLOS ONE

Reviewers' comments:

# Editor report: Acceptance letter

**DOI:** 10.1371/journal.pone.0351659.r004

**Authors:** Morufu Raimi

PONE-D-26-17339R1

PLOS One

Dear Dr. Asher,

I'm pleased to inform you that your manuscript has been deemed suitable for publication in PLOS One. Congratulations! Your manuscript is now being handed over to our production team.

At this stage, our production department will prepare your paper for publication. This includes ensuring the following:

* All references, tables, and figures are properly cited

* All relevant supporting information is included in the manuscript submission,

* There are no issues that prevent the paper from being properly typeset

You will receive further instructions from the production team, including instructions on how to review your proof when it is ready. Please keep in mind that we are working through a large volume of accepted articles, so please give us a few days to review your paper and let you know the next and final steps.

Lastly, if your institution or institutions have a press office, please let them know about your upcoming paper now to help maximize its impact. If they'll be preparing press materials, please inform our press team within the next 48 hours. Your manuscript will remain under strict press embargo until 2 pm Eastern Time on the date of publication. For more information, please contact onepress@plos.org.

You will receive an invoice from PLOS for your publication fee after your manuscript has reached the completed accept phase. If you receive an email requesting payment before acceptance or for any other service, this may be a phishing scheme. Learn how to identify phishing emails and protect your accounts at https://explore.plos.org/phishing.

If we can help with anything else, please email us at customercare@plos.org.

Thank you for submitting your work to PLOS ONE and supporting open access.

Kind regards,

PLOS ONE Editorial Office Staff

on behalf of

Prof Morufu Olalekan Raimi

Academic Editor

PLOS One
